# Effects of Probiotics and Wheat Bran Supplementation of Broiler Diets on the Ammonia Emission from Excreta

**DOI:** 10.3390/ani11092703

**Published:** 2021-09-15

**Authors:** Nikoletta Such, Gábor Csitári, Petra Stankovics, László Wágner, Ilona Anna Koltay, Valéria Farkas, László Pál, Patrik Strifler, Károly Dublecz

**Affiliations:** 1 Institute of Physiology and Nutrition, Georgikon Campus, Hungarian University of Agriculture and Life Sciences, Deák Ferenc Street 16, 8360 Keszthely, Hungary; such.nikoletta.amanda@phd.uni-mate.hu (N.S.); csitari.gabor@uni-mate.hu (G.C.); wagner.laszlo@uni-mate.hu (L.W.); ilcsu92@gmail.com (I.A.K.); farkas.valeria@uni-mate.hu (V.F.); pal.laszlo@uni-mate.hu (L.P.); strifler.patrik@phd.uni-mate.hu (P.S.); 2 Institute of Crop Production, Georgikon Campus, Hungarian University of Agriculture and Life Sciences, Deák Ferenc Street 16, 8360 Keszthely, Hungary; stankovics.petra@gmail.com

**Keywords:** urease activity, ammonia, broiler, nitrogen, wheat bran, *Lactobacillus farciminis*, *Clostridium butyricum*

## Abstract

**Simple Summary:**

Animal production is the main source of ammonia emission worldwide and all member countries of the European Union must reduce their national emissions. Among nutritional strategies, feeding low protein diets, using more nutritional phases, or using different feed additives can decrease the nitrogen excretion of animals and, in this way, lower the ammonia volatilisation from the manure. Pro- and prebiotics are widely used to improve gut health and to decrease the incidence of diseases. Numerous research findings have been published on the practical effects of pro- and prebiotics, but their impact on the urinary and faecal N excretion in chickens has not been completely clarified yet. In this research, the effects of using lactic acid and butyric acid producing bacterial strains, and wheat bran as a potential prebiotic, was tested with broiler chickens. Both probiotics increased the dry matter content and decreased the urinary N ratio of the excreta, which is positive from an ammonia emission point of view. Wheat bran and its xylan-oligosaccharides decreased both the ammonium -N content and the urinary N ratio. The results proved that beside the well-known nutritional techniques, the feed additives, which modify the gut microbiota and the fermentation in the caeca, can decrease the urinary-N excretion, and in this way lower the ammonia emission of broiler chickens.

**Abstract:**

Ammonia emission is a concern for the poultry industry from both environmental and animal welfare points of view. The objective of this research was to determine whether probiotics or wheat bran supplementation of broiler diets can modify the N composition of the excreta and the dynamics of ammonia volatilisation emission from the manure. A total of 120-day-old Ross 308 broiler chickens were fed six different diets. The treatments included a corn and soybean meal-based control diet (C) and diets containing wheat bran (WB). Both diets were fed alone and with supplementation of a lactic acid (*Lactobacillus farciminis,* LAB) and a butyric acid (*Clostridium butyricum, BAB*) producing bacterial strain. Treatment BAB had a significant effect on the dry matter content of the excreta and both probiotics decreased the amount of excreted uric acid. Treatment WB resulted in a significantly lower NH^+^_4_-N concentration of excreta and a tendency toward reduced uric acid content. Treatment LAB reduced the urinary N ratio of excreta. Among dietary treatments, WB resulted in the highest urease producing cell numbers in the excreta, but this difference was not significant. Based on our results, similar to pigs, the soluble fibre fraction of poultry diets can also modify the urinary to faecal N ratio of the excreta.

## 1. Introduction

Antibiotics have been used in animal husbandry as growth promoters for more than 60 years [1]. However, their application has led to the increase in antibiotic-resistant bacteria. This has come to the forefront of attention as a potential source of danger in recent years [2]. The use of antibiotics as a growth promoter was banned in the European Union in 2006, and since then, several alternative feed additives have been tested, including pre- and probiotics [3]. Probiotics are defined by the World Health Organization as “microorganisms” administered in a live form and in adequate amounts to improve the health of the host. Probiotics can kill pathogenic microorganisms with the help of their antimicrobial compounds such as bacteriocins and organic acids. They improve the stability of the gastrointestinal microbial environment, thereby preventing the binding and colonization of pathogens, stimulating intestinal-induced immune responses, improving digestion and absorption of nutrients [4]. Prebiotics are mostly oligosaccharides, supplying nutrients for bacteria considered as favourable [5]. Oligosaccharides form because of fibre degradation and are primary substrates for the growth of intestinal microorganisms [6]. Fermentable oligosaccharides increase the concentration of short chain fatty acids (SCFA) in the cecum and colon, which decrease the pH and hence the colonisation of the potentially harmful bacteria [7]. Beside the well-known prebiotics such as fructose oligosaccharides and mannan oligosaccharides, the soluble arabinoxylan of wheat also has a prebiotic effect if the diets are supplemented with exogenous xylanase [8]. SCFAs also play an important role as an energy source of the host and the epithelial cells. Acetic acid, propionic acid, butyric acid and lactic acid are also important in the “cross-feeding” mechanism of the different bacterial groups [9]. The more intensive bacterial fermentation increases bacterial biomass, bacterial diversity and hence the excreted microbiota [10]. The non-digestible and unutilized dietary nitrogen excreted via faeces and urine is converted to gaseous ammonia by aerobic or anaerobic bacteria in the manure [11]. Much of the ammonia released from manure comes from the hydrolysis of urea [12], or in the case of birds from the breakdown of uric acid [13].

It is well known that in mammals such as pigs, the urinary N content can be decreased if fermentable carbohydrates, for example sugar beet pulp, are fed. The increased bacterial biomass can reduce ammonia emission in the intestines, because ammonia formed in the post-small intestinal tract is converted in a greater proportion to bacterial protein [14]. As a result, less ammonia is absorbed from the caeca and recirculated to the liver, which reduces the synthesis of uric acid. Bacterial proteins are excreted via faeces, and the bacteria in the manure are unable to release ammonia from this protein in a short time. Since the fibre fermentation capacity of birds is limited, this mechanism is not yet proved in the case of chickens [15]. 

Based on the available research results, uric acid represents 50–60% of the total N content of poultry excreta [16]. With the development of nutrition and genetics, this ratio may have changed, but only a few results are available concerning this topic in the literature. Ammonia emission is a concern for the poultry industry from both an environmental and animal welfare point of view. Potential effects on birds include respiratory diseases, viral infections, decreased production, and higher mortality [17].

Numerous research findings have been published on the practical effects of probiotics [18], but their impact on the urinary and faecal N excretion in chickens has not been completely clarified yet. Probiotic bacteria can modify protein digestibility and the proteolytic breakdown of the undigested protein in the hind gut segments [19].

The results of studies evaluating biological agents to reduce ammonia emissions from poultry litter are contradictory [20,21,22] and the reason for the reduction in ammonia emissions has not been completely clarified either. 

In our previous studies, it has been shown that no differences were observed in growth parameters among the treatment groups, although wheat bran had a beneficial effect on the caecal microbiota composition of broiler chickens. It can increase the ratio of butyric acid, decrease the pH, and decrease the abundance of *Campylobacter jejuni* [8]. Feeding wheat bran also increases the growth of the bacteria responsible for mucosal regeneration, for example *Akkermansia muciniphila* [23].

In this study, the effect of using wheat bran, containing arabinoxylan, as a potential prebiotic and two existing probiotics were used to find out their effects on the N-composition of excreta, on the dynamics of in vitro ammonia emission, and on the amounts of ureolytic faecal bacteria.

## 2. Materials and Methods

A floor pen trial was carried out at the experimental farm of the Institute of Physiology and Nutrition, Hungarian University of Agriculture and Life Sciences (Georgikon Campus, Keszthely, Hungary). The animal experiment was approved by the Institutional Ethics Committee (Animal Welfare Committee, Georgikon Campus, Hungarian University of Agriculture and Life Sciences). One hundred and twenty-one-day-old Ross 308 broiler chickens were purchased from a commercial hatchery (Gallus Ltd. Devecser, Hungary) and placed into six floor pens, 20 chickens per pen (10 chickens per m^2^). Beside a corn and soybean-based control diet (C), a diet containing wheat bran was used (WB). Both diets were fed with or without probiotics. The probiotics used contained lactic acid-producing (LAB; *Lactobacillus farciminis CNMA67-4R*, Biacton, 5 × 10^9^ CFU/kg) and butyric acid-producing bacteria (BAB; *Clostridium butyricum CBM 588;* Miya-Gold, 2.5 × 10^9^ CFU/kg). The inclusion rate of both products was 0.5 g/kg. So, the dietary treatment combinations were: C, WB, C + LAB, C + BAB, WB + LAB, WB+BAB. The arrangement of the treatments was a 2 × 3 factorial design with two different diets (C, WB) and three probiotic treatments (LAB, BAB, Ø). Starter diets were fed from day 1 to 10, grower diets from day 11 to 24, and finisher diets from day 25 to 40. Feed and drinking water were available ad libitum for the animals. The composition and nutrient content of diets are shown in Table 1 and Table 2. As it can be seen in Table 1, the wheat bran content of the starter diets was 3% and those of the grower and finisher diets were 6%, respectively. Control diets contained slightly more soybean meal and the lower energy content of wheat bran was compensated by more oil incorporation. The crude protein and AMEn contents of the diets were close to each other. The crude fibre content of the WB diets was about 0.5% higher (Table 2). Computer controlled housing and climatic conditions were maintained during the trial according to the breeder’s recommendations [24].

At day 37, eight chicks, with similar body weight, were transferred from each pen to individual balance cages, and excreta samples were collected, after 3 days adaptation period, on day 40. In this phase, birds were fed the same finisher diets as before, in pens. At day 40 a minimum of 200 g excreta was collected from each animal and stored in a refrigerator at −20 °C. Before the analyses, excreta were homogenized properly, then the pH, dry matter content, total N, ammonium-N (NH_4_^+^-N) and uric acid-N contentsdetermined. The total N was determined according to the Kjeldahl method by the Foss-Kjeltec 8400 Analyzer Unit [25], the ammonium-N by the method of Peters [26], and the uric acid-N as described by Marquardt [27]. The sum of ammonium-N and uric acid-N was considered as urinary N content [28]. The in vitro ammonia emission measurement was carried out at two time points using the method of Santoso [13]. The ammonia concentration of the air was measured with Draeger equipment (model X-am 5600; Drägerwerk AG & Co. KGaA, Lübeck, Germany). Samples were thawed 19 h before measurement. A total of 50 g homogenised excreta samples were placed into 1L double-sealed containers. The samples were placed in the tanks at a temperature of 20 °C. Each container was equipped with a cover containing a hole to allow insertion of a gas measuring tube that was sealed inside with adhesive plaster. Measurements were taken two times: 1.5 h and then 4 h after entering the tank. The ammonia measurement range of the equipment’s sensor was 0–300 ppm. The adhesive plaster was punctured, and 1000 mL of headspace air was collected from approximately 10 cm above the sample surface. After sampling, the tubes were sealed again. The concentration of NH_3_ was expressed as milligrams per liter.

The number of urease enzyme producing bacteria of excreta was estimated using the Most Probable Number (MPN) method based on the work of Fujita [29] using the Urea Broth Base (Scharlab). For each of the triplicate samples 10-fold serial dilutions (10^−1^–10^−8^) were prepared in sterile phosphate buffered saline. The assay was performed using microplates. The microplates were incubated at room temperature for three days before being scored as positive or negative for urea hydrolysis. The color change due to the reaction was recorded with a fluorescence microplate reader (Perkin Elmer Enspire 2300, PerkinElmer, Inc; Waltham, MA, USA). Unfortunately, two groups of samples (WB+BAB, WB+LAB) failed due to experimental error and could not be evaluated.

Statistical evaluation was performed using SPSS 24.0 software. Two-way ANOVA was used in a 2 × 3 factorial arrangement. The model included effects of diet, probiotics, and the interaction between them. To understand the significant interactions between the main factors in the ammonia emission results, the treatment effects were also evaluated by one-way ANOVA. The experimental unit for the analysis of the fresh excreta was a cage with one individual bird. The Kruskal–Wallis test was used to evaluate the ratio of the fecal and urinary N. The results on the ureolytic bacteria were analyzed by one-way ANOVA. The probability level of *p* ≤ 0.05 was considered as statistically significant.

## 3. Results

Neither the diet composition nor the probiotic treatments affected the weight gain, feed intake, and fed conversion of chickens. The production traits results of the trial have been published earlier [30].

### 3.1. Nitrogen Forms

The dietary treatments did not significantly affect the amount of total N excretion. After feeding the control and wheat bran containing diets, statistically significant differences were found only in the NH^+^_4_-N content of the excreta (*p* = 0.024). WB treatment resulted in lower NH^+^_4_-N concentrations (Table 3). LAB tended to increase (*p* < 0.099) and the BAB significantly increased (*p* = 0.012) the dry matter content compared with the control treatment. There was no significant interaction between the two main factors at any parameters.

**Figure 1:**
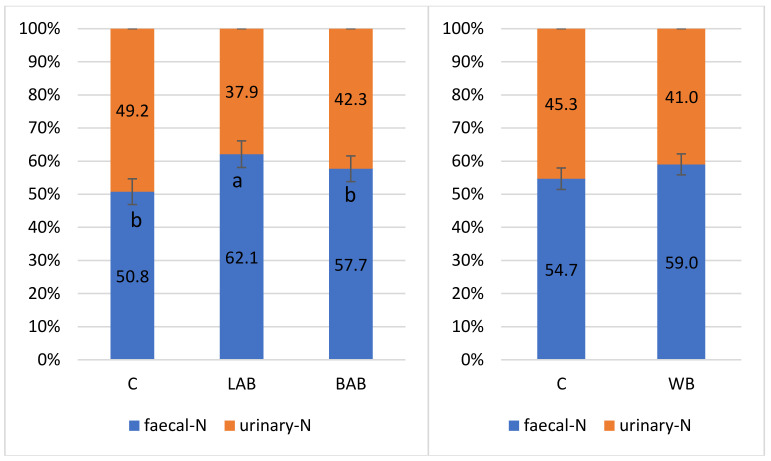
Treatment effects on the ratio of the faecal and urinary N. ^a,b^ means with different superscripts are significantly different.

Figure 1 shows that both probiotics increased the faecal and decreased the urinary N content of the excreta, but only the difference between LAB and control treatments was significant. Compared to the control group, the differences were 11% and 7% in the LAB and BAB treatments respectively. Feeding the wheat bran containing diets failed to modify the faecal and urinary N ratio.

### 3.2. NH_3_ Release from Excreta

Dietary treatments did not influence NH_3_ release from the excreta significantly after 1.5 h, but both the probiotics and wheat bran increased the volatilisation numerically (Table 4). After 4 h, however, the wheat bran effect was already significant. At 1.5 h significant probiotic and wheat bran interaction was observed. The reason for this was that LAB increased the volatilisation, adding to the control diet, while the opposite was found when it was given to the WB diet.

### 3.3. Number of Ureolytic Bacteria in Excreta

**Figure 2:**
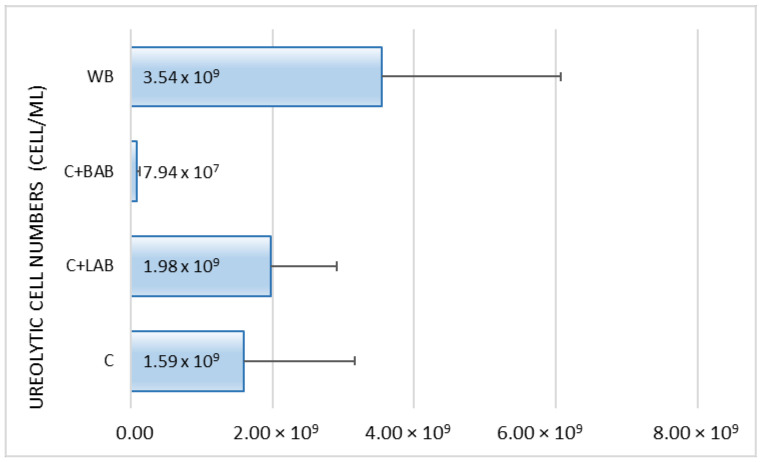
Number of ureolytic bacteria of the excreta.

Although big differences were found between the averages, these differences were not significant because of the high standard error values (Figure 2). However, it can be seen that the number of urease producing bacteria in the WB group was about twice as high as in the control or LAB treatment groups. Treatment C+BAB decreased this parameter.

## 4. Discussion

Several directives force the reduction of ammonia emissions from the agricultural sector (Common Agricultural Policy (CAP), the Nitrate Directive (91/676/EEC), and the Water Framework Directive (2000/60/EC)). The new NEC Directive ensures 2020 and 2030 emission reduction commitments for five main air pollutants, including NH_3_. The directive requires that the EU Member States draw up National Air Pollution Control Programmes. In Hungary, the application procedure is in progress, and nutrition of farm animals is a key factor in reducing ammonia emission.

Similarly, as in the findings of Jeong et al. [31] who fed *B. subtilis* supplemented diets to broiler chickens, no significant differences were observed in this trial in the uric acid-N, and total N content of excreta. However, in our study, the amount of NH^+^_4_-N in the excreta of chickens fed the bran containing diet decreased significantly. Santos et al. [32] obtained similar results for ammonium-N reduction, after feeding rice and soybean husks with broilers.

Probiotics increased the dry matter content of the excreta in our case. In a study with horses [33], a similar result was obtained regarding the dry matter content of faeces after probiotic treatment. Ammonia formation in barns is directly regulated by factors such as pH, temperature, and litter moisture content [34]. An increase in the dry matter content of the excreta has a positive effect on litter quality, which can reduce the incidence of foot pad lesions, as well as greatly influence the release of ammonia [35]. In our trial probiotics decreased the ratio of urinary N of the excreta. The mechanism behind this result could be that both butyric and lactic acid decrease the pH, and in this way inhibit the protease activity in the caeca. This can lead to increased incorporation of amino acids and ammonia by the microbiota, which leads to increased N content of the faeces and reduced renal N-excretion [36,37,38]. Lower urinary N excretion could mean lower water intake and increased dry matter content of the excreta [39]. No such interaction has been published yet in the case of broiler chickens. 

In poultry, faeces and urine are excreted together in a mixture, making it difficult to separate faecal and urinary nitrogen. O’dell et al. [27] found that the sum of uric acid and NH^+^_4_-N of the excreta gives approximately the total amount of urinary nitrogen in birds. Uric acid is more easily converted to ammonia than ammonium-N. This is also affected by pH, since in acidic pH, most of the ammonia is converted to ammonium ions, which volatilize slowly [14]. Dietary fibre results increased SCFA production in the caeca, and this modified the pH. This potential pH change in the caecal content did not affect the pH of excreta [40].

In our study, only treatment LAB increased the ratio of faecal N and decreased the ratio of urinary N in the excreta. Wheat bran in this trial failed to affect this parameter. It suggests that, contrary to pigs [14], the proportion of N-containing substances excreted via faeces and urine are more constant in poultry. Roberts et al. [41] showed that layer diets supplemented with DDGS (Distillers Dried Grains with Solubles), wheat middlings, or soybean hulls decreased ammonia emission from the manure but does not affect the ratio of urinary and faecal N of the excreta. In that experiment, the fibre sources were mostly structural. Since birds have relatively short large intestines and a quicker passage rate, it means a shorter period for bacterial fermentation. Because only the soluble and small fibre fractions can reach the caeca, the effects of caecal fermentation on the N composition of excreta did not change in that trial. As far as the authors know, it is the first result which proves that fermentable carbohydrates can also push the urinary N ratio towards faecal N in poultry species.

In contrast to previous studies [13,31,42,43], the type of probiotics used in our experiment did not have a statistically significant effect on the amount of ammonia released from the excreta. However, wheat bran supplementation resulted in significantly increased ammonia emission in vitro. Increased ammonia emissions due to wheat bran may have been caused by metabolic changes in the bacterial flora. In our previous study using the 16S rRNA sequencing method, the diversity of the bacterial flora of the caecum did not change significantly under the influence of wheat bran [22,44], but several differences were observed at genus level. In the study of Vermeulen et al. [9], wheat bran supplementation significantly increased caecal microbiota abundance. Kieffer et al. [45] found that the metabolic change of the bacterial flora may provide more relevant information than the quantitative change in the microbiota. Stanley et al. [46] showed that the microbial composition of faeces in poultry was not the same as that of caecal microbiota. In the present study the xylan-oligosaccharides, the products of enzymatic breakdown of arabinoxylans of wheat can influence the amount and composition of the caecal microbiota and hence influence the urease activity of the manure.

Although the differences were not significant, the number of ureolytic bacterial flora in the excreta was two times higher in the WB group, which could have played a role in the in vitro NH_3_ emission. Many viable intestinal organisms such as *Bacteroides, Bifidobacteria, Clostridia, Proteus* spp., and *Klebsiella* spp. have urease activity. Dietary probiotics have been shown to suppress the growth of urease-producing bacteria [47], either by producing antimicrobials or by reducing the pH, and subsequently reducing ammonia levels in chicken caeca [48]. According to Ahmed [49] the *Bacillus amyloliquefaciens* reduce urease-generating microbiota in the gastrointestinal lumen.

## 5. Conclusions

Pre- and probiotics are widely used to support gut health and the beneficial intestinal flora. Based on our results, these feed supplements can also modify the N-composition of excreta, which is currently a less researched area. From this study, it can be concluded that probiotics increase the dry matter content of the excreta, probably because of the lower urinary nitrogen excretion, and, therefore, the lower water intake of the chickens. The addition of wheat bran to the diets did not affect the amount of N excreted via faeces and urine. This result proves that, in contrast to mammals, fermentable fibres in poultry have only limited effect on the proportion of the excreted bacterial protein. In poultry, this mechanism differs from the microbial processes in pigs, because in poultry species only a small proportion of undigested fibres and oligosaccharides enter the caeca, and the content of it represents a relatively small proportion of the whole excreta. On the other hand, based on these results, the soluble fibre fraction of the feed can influence the amount of NH_3_ released from the excreta and can change the dynamics of the emission.

## Figures and Tables

**Table 1:** Composition of experimental diets (g/kg as fed).

	Starter Day 1–10		Grower Day 11–24		Finisher Day 25–40	
	C	WB	C	WB	C	WB
Maize	466	434	534	469	589	524
Wheat bran	0	30	0	60	0	60
Extracted soybean meal	338	333	361	352	310	300
Sunflower meal	80	80	0	0	0	0
Sunflower oil	63	70	62	76	60	74
Limestone	19	19	15	15	15	15
MCP	15	15	14	14	13	13
L-lysine	5	5	2	2	2	2
DL-methionine	4	4	3	3	3	3
L-threonine	1	1	1	1	0	1
L-valine	1	1	0	0	0	0
NaCl	3	3	3	3	3	3
NaHCO_3_	1	1	1	1	1	1
Premix ^1^	4	4	4	4	3.5	3.5
Phytase ^2^	0.1	0.1	0.1	0.1	0.1	0.1
NSP enzyme ^3^	0.1	0.1	0.1	0.1	0.1	0.1
SUM	1000	1000	1000	1000	1000	1000

Abbreviations: C—control; WB—corn-soybean-based diet supplemented with 30, 60 and 60 g/kg wheat bran in the starter, grower, and finisher diets, respectively; ESM—extracted soybean meal; MCP—monocalcium phosphate; ^1^ Premix was supplied by UBM Ltd. (Pilisvörösvár, Hungary). The active ingredients contained in the premix were as follows (per kg of diet): Starter and grower premixes—retinyl acetate—5.0 mg, cholecalciferol—130 g, dl-alpha-tocopherol-acetate—91 mg, menadione—2.2 mg, thiamine—4.5 mg, riboflavin—10.5 mg, pyridoxin HCl—7.5 mg, cyanocobalamin—80 g, niacin—41.5 mg, pantothenic acid—15 mg, folic acid—1.3 mg, biotin—150 g, betaine—670 mg, monensin-Na—110 mg (only grower), narasin—50 mg (only starter), nicarbazin—50 mg (only starter), antioxidant—25 mg, Zn (as ZnSO_4_H_2_O)—125 mg, Cu (as CuSO_4_5H_2_O)—20 mg, Fe (as FeSO_4_H_2_O)—75 mg, Mn (as MnO)—125 mg, I (as KI)—1.35 mg, Se (as Na_2_SeO_3_)—270 g; Finisher premix—retinyl acetate—3.4 mg, cholecalciferol—97 g, dl-alpha-tocopherol-acetate—45.5 mg, menadione—2.7 mg, thiamin—1.9 mg, riboflavin—5.0 mg, pyridoxin HCl—3.2 mg, cyanocobalamin—19 g, niacin—28.5 mg, pantothenic acid—10 mg, folic acid—1.3 mg, biotin—140 g, L-ascorbic acid—40 mg, betaine—193 mg, antioxidant—25 mg, Zn (as ZnSO_4_H_2_O)—96 mg, Cu—9.6 mg, Fe (as FeSO_4_H_2_O)—29 mg, Mn (as MnO)—29 mg, I (as KI)—1.2 mg, Se (as Na_2_SeO_3_)—350 g. ^2^ Phytase was Quantum Blue^®^ (AB Vista, Marlborough, UK). ^3^ NSP (non-starch polysaccharide) degrading enzyme Econase XT^®^ (AB Vista, Marlborough, UK).

**Table 2:** Measured nutrient contents of the experimental diets (g/kg as fed).

	Starter Day 10		Grower Day 24		Finisher Day 40	
	C	WB	C	WB	C	WB
AME_n_ (MJ/kg)	12.1	12.2	13.1	13.0	13.0	13.1
Dry matter	88.8	89.0	88.5	88.8	88.2	88.8
Crude protein	22.9	23.0	20.7	21.2	18.8	19.1
Crude fat	8.3	9.2	9.1	10.1	8.9	10.0
Crude fiber	4.02	4.575	3.77	4.18	3.63	4.33
Ash	6.69	6.83	5.61	5.96	5.43	5.69
Ca	1.07	1.08	0.94	0.94	0.89	0.89
P	0.80	0.81	0.67	0.71	0.66	0.7
Starch	30.5	29.4	36.9	33.6	38.7	36.4

**Table 3:** Composition and pH of excreta samples.

Treatments	Total N	NH^+^_4_-N	Uric Acid-N	pH	Dry Matter
	mg/g DM				%
Probiotic effects					
C	50.076	4.864	17.531	6.322	21.454 ^a^
LAB	51.576	4.806	14.143	6.142	25.568 ^ab^
BAB	50.468	4.065	15.407	6.278	27.321 ^b^
Diet effects					
C	50.624	5.172 ^a^	16.836	6.258	24.315
WB	50.75	4.000 ^b^	14.664	6.242	25.358
Pooled SEM	2.744	0.271	0.846	0.039	0.857
*p*-values					
Probiotic	0.979	0.273	0.224	0.168	0.012
Diet	0.992	0.024	0.226	0.915	0.313
Probiotic × Diet	0.827	0.128	0.875	0.170	0.412

^a,b^ means with different superscripts of the same column are significantly different.

**Table 4:** Treatment effects on the in vitro NH_3_ emission.

Treatments		1.5 h	4 h
C		4.80	33.10
	LAB	10.33	40.66
	BAB	5.08	32.91
WB		8.71	42.85
	LAB	4.16	41.66
	BAB	10.55	45.44
SEM		0.867	1.765
Probiotic effects			
C		6.31	36.93
LAB		6.98	39.92
BAB		7.63	37.81
Diet effects			
C		6.50	34.08 ^a^
WB		7.40	42.36 ^b^
Pooled SEM		0.89	1.85
*p*-values			
Probiotic		0.805	0.803
Diet		0.593	0.029
Probiotic × Diet		0.048	0.493

^a,b^ means with different superscripts in the same column are significantly different.

## Data Availability

All data generated or analyzed during this study are included in this published article.
